# Whole transcriptome analysis revealed the regulatory network and related pathways of non-coding RNA regulating ovarian atrophy in broody hens

**DOI:** 10.3389/fvets.2024.1399776

**Published:** 2024-05-29

**Authors:** Hanlin Xiong, Wendong Li, Lecong Wang, Xuchen Wang, Bincheng Tang, Zhifu Cui, Lingbin Liu

**Affiliations:** College of Animal Science and Technology, Southwest University, Chongqing, China

**Keywords:** broodiness, ovarian development, whole transcriptome analysis, ncRNAs, ceRNA network

## Abstract

Poultry broodiness can cause ovarian atresia, which has a detrimental impact on egg production. Non-coding RNAs (ncRNAs) have become one of the most talked-about topics in life sciences because of the increasing evidence of their novel biological roles in regulatory systems. However, the molecular mechanisms of ncRNAs functions and processes in chicken ovarian development remain largely unknown. Whole-transcriptome RNA sequencing of the ovaries of broodiness and laying chickens was thus performed to identify the ncRNA regulatory mechanisms associated with ovarian atresia in chickens. Subsequent analysis revealed that the ovaries of laying chickens and those with broodiness had 40 differentially expressed MicroRNA (miRNAs) (15 up-regulated and 25 down-regulated), 379 differentially expressed Long Noncoding RNA (lncRNAs) (213 up-regulated and 166 down-regulated), and 129 differentially expressed circular RNA (circRNAs) (63 up-regulated and 66 down-regulated). The competing endogenous RNAs (ceRNA) network analysis further revealed the involvement of ECM-receptor interaction, AGE-RAGE signaling pathway, focal adhesion, cytokine-cytokine receptor interaction, inflammatory mediator regulation of TRP channels, renin secretion, gap junction, insulin secretion, serotonergic synapse, and IL-17 signaling pathways in broodiness. Upon further analysis, it became evident that *THBS1* and *MYLK* are significant candidate genes implicated in the regulation of broodiness. The expression of these genes is linked to miR-155-x, miR-211-z, miR-1682-z, gga-miR-155, and gga-miR-1682, as well as to the competitive binding of novel_circ_014674 and MSTRG.3306.4. The findings of this study reveal the existence of a regulatory link between non-coding RNAs and their competing mRNAs, which provide a better comprehension of the ncRNA function and processes in chicken ovarian development.

## Introduction

Broodiness, a natural maternal behavior observed in chickens, turkeys, and geese, is characterized by appetite loss and a halt in egg-laying and incubation activities. This phenomenon poses a challenge to the progress of the modern poultry industry. Generally, broodiness is initiated by a signal that stops egg production (1). The synchronization of hormones secreted by the hypothalamic-pituitary-gonadal (HPG) axis is a prerequisite for the maintenance of broodiness (2). Studies postulate that the ovary is larger in weight and volume during the laying stage than during the broodiness stage. The ovarian weight of the egg-laying chickens was 48.17 g, significantly higher than the 2.67 g weight observed in broody chickens. The upper-grade follicles in the ovary are in active development during the laying stage, while the inner of the ovary is composed of atrophic and atretic follicles during the broodiness stage (3, 4). The eukaryotic genome has a much lower gene density compared to the prokaryotic genome, reflecting an efficient evolutionary adaptation to meet its complex biological demands. Both protein-coding and non-protein-coding RNA play a role in transcriptional regulation. The human genome transcribes over 90% of its content. As a result, the majority of transcribed genes generate noncoding RNAs (ncRNAs), which have primarily function as regulatory elements (5).

Analysis of RNA sequences during DNA transcription reveals that coding RNAs, responsible for protein translation, make up only approximately 4% of total RNA. Conversely, ncRNAs are widely distributed and play diverse roles in gene regulation (6). Both protein-coding and non-protein-coding RNA play a role in transcriptional regulation. Notably, most of the transcriptome comprises ncRNAs (7). Several studies postulate that non-coding RNAs (miRNA, lncRNA, lncRNA, siRNA) play a role in controlling ovarian development and performance. In particular, miR-182 and miR-15a have been reported to modulate granulosa cell proliferation and apoptosis by altering hormone production (8, 9). LncRNAs also serve as regulators in the reproductive process of poultry. Noteworthy, Mao et al. (10) discovered 24,601 lncRNAs linked to egg production in domestic pigeons. Over the last few years, circRNA has become a popular topic in RNA research because of its involvement in the regulation of multiple molecules in organisms through post-transcriptional regulation of classical signaling pathways (11). CircDDX10 is associated with ovarian aging, circTCP11 with pig litter size, and chi_circ_0008219 with goat reproductive rate (12–14). Despite the significance of ncRNA as vital regulators in many biological processes, their molecular regulatory mechanisms during broodiness of Chengkou mountain chicken remain unknown.

Chengkou mountain chicken is a famous local chicken variety in China, mainly reared in Chongqing municipality. However, it has a low production efficiency and high breeding costs because of its strong broodiness ability (around 90%), low egg production, and slow early growth rate. This study aimed to identify and analyze the differentially expressed non-coding RNAs and their expression patterns of Chengkou mountain chicken with a normal laying ovary and ovary with broodiness using whole-transcriptome RNA-seq. The findings of this study provide insights into the molecular processes that control the broodiness of Chengkou mountain chicken, which lay a basis for the utilization of marker-assisted selection and genetic improvement of chicken.

## Materials and method

### Animal and tissue samples

The experimental animals were 6 chickens from the same batch obtained from the Chengkou Mountain Chickens Genetic Research Institute (Chongqing, China). The Chengkou mountain chickens exhibits favorable germplasm traits, including robust foraging ability, resistance to coarse feeding, suitability for wild grazing and free range, strong disease resistance, and high adaptability. Their egg production and broody situation were recorded daily. Three individuals exhibiting broody behavior and entering the classic broody phase 30 days earlier were chosen. Their ovarian tissues were harvested in liquid nitrogen for RNA and transcriptome sequencing and then stored at −80°C awaiting RNA extraction.

### Chicken ovary histomorphology

The histological characteristics of chicken ovaries were evaluated through hematoxylin and eosin staining, following paraffin embedding of the samples and sectioning. The histological ovary micromorphology was visualized using a microscope (Olympus IX53, Japan), followed by image capture using the microphotographic system (Olympus DP71, Japan). The ultrastructure of the chicken ovaries was observed using a transmission electron microscope (TEM Hitachi HT7800/HT7700).

### RNA isolation, library construction, and sequencing

The data quality of RNA Q30 in each sample was more than 92.30%, indicating that the sequencing data quality was good (Supplementary material 4). The total RNA of the ovary tissues was extracted using the Trizol reagent kit (Invitrogen, Carlsbad, CA, United States) following the manufacturer’s instructions. RNA quality was assessed using an Agilent 2100 Bioanalyzer (Agilent Technologies, Palo Alto, CA, United States) and RNase-free agarose gel electrophoresis. After total RNA was extracted, rRNAs were removed to retain mRNAs and ncRNAs. The enriched mRNAs and ncRNAs were fragmented into short fragments by using fragmentation buffer and reverse transcribed into cDNA with random primers. Second-strand cDNAs were synthesized using DNA polymerase I, RNase H, dNTPs (dUTP instead of dTTP), and buffer. The cDNA fragments were then purified using the QiaQuick PCR extraction kit (Qiagen, Venlo, The Netherlands), end-repaired, poly(A) added, and ligated to Illumina sequencing adapters. The second-strand cDNAs were digested using UNG (uracil-N-glycosylase), after which they were size selected by agarose gel electrophoresis, PCR amplified, and sequenced using Illumina HiSeqTM 4000 (Gene Denovo Biotechnology Co, Guangzhou, China). Then the 3′ adapters were added and the 36–48 nt RNAs were enriched. The 5′ adapters were then ligated to the RNAs as well. The ligation products were reverse transcribed through PCR amplification, and the 140–160 bp size PCR products were enriched to generate 6 cDNA libraries, which was sequenced using Illumina HiSeq Xten (Gene Denovo Biotechnology Co, Guangzhou, China).

### Identification and analysis of miRNA, lncRNA and circRNA

To refine the raw data, we exclude reads with low quality (*Q*-value ≤20), reads that include adapters, reads shorter than 18 nt, and reads featuring polyA (15). The clean tags were then aligned with small RNAs in the GeneBank database (Release 209.0) and Rfam database (Release 11.0) to identify and remove rRNA, scRNA, snoRNA, snRNA, and tRNA. All of the clean tags were then searched against miRBase database (Release 22) to identify known (Species studied) miRNAs (exist miRNAs). After tags were annotated as mentioned previously, the annotation results were determined in this priority order: rRNA etc. > exist miRNA > exist miRNA edit > known miRNA > repeat > exon > novel miRNA > intron. The tags that cannot be annotated as any of the above molecules were recorded as unann.

Filtration of the raw data involved removing reads of low quality (*Q*-value ≤20), reads with adapters, reads composed entirely of A bases, and reads containing more than 10% unknown nucleotides. Following this, alignment of the clean data with chicken rRNA sequences using Bowtie2 facilitated the removal of reads that aligned with rRNA (16). Softwares CNCI (17) (version 2) and CPC (18) (version 0.9-r2)^1^ and FEELNC (19) (version v0.2)^2^ were used to assess the protein-coding potential of the novel transcripts using their default parameters. The final prediction for lncRNAs resulted from the intersection of three software programs.

The short reads alignment tool Bowtie2 (16) (version 2.2.8) was thus used to map the reads to the ribosomal RNA (rRNA) database. The rRNA-mapped reads were removed, and the remaining reads were further used for other alignments and analyses. The rRNA-removed reads from each sample were then mapped to the reference genome using HISAT2 (20) (version 2.1.1). Reads that mapped to the reference genomes were discarded, while the unmapped reads were used for circRNA identification. Anchor reads that aligned in the reversed orientation (head-to-tail) indicated circRNA splicing and were subjected to find_circ to identify the circRNAs (21). We identified ncRNAs with fold change ≥2 and FDR (false discovery rate) <0.05 in a comparison as significant DE ncRNAs.

### Construction and visualization of the ceRNA network

The ceRNA network was constructed based on the ceRNA theory as follows: (1) the target relationship between miRNA and candidate ceRNA and the negative correlation of expression, (2) the positive correlation between the expression levels of candidate ceRNAs, and (3) enrichment degree of candidate ceRNA binding to the same miRNA. miRNA-target gene pairs were first identified because ceRNAs are mutually regulated by miRNAs. The target genes for differential miRNAs were then predicted as the first step in the study of ceRNA regulatory networks. The expression correlation between mRNA-miRNA, lncRNA-miRNA, and circRNA-miRNA was then evaluated using the Spearman rank correlation coefficient (SCC). Pairs with SCC <−0.7 were selected as negatively co-expressed lncRNA-miRNA pairs, mRNA-miRNA pairs, or circRNA-miRNA pairs. The mRNAs, lncRNAs, and circRNAs were miRNA target genes and were all differentially expressed. The expression correlation between lncRNA-mRNA and circRNA-mRNA was evaluated using the Pearson correlation coefficient (PCC). Pairs with PCC >0.9 were selected as co-expressed lncRNA-mRNA pairs or circRNA-mRNA pairs. Both the mRNA and lncRNA or mRNA and circRNA in these pairs were targeted and co-expressed negatively with a common miRNA. A hypergeometric cumulative distribution function test was then done to test whether the common miRNA sponges between the two genes were significant. Only the gene pairs with a *p*-value less than 0.05 were selected. For each gene pair (A, B), we denoted all their regulator miRNAs as miRNA set C (regulating gene A) and D (regulating gene B). Where *x* stands for the number of common miRNAs that regulate both genes, *U* is the number of all the miRNAs in the work, *M* is the size of miRNA set C, and *N* is the size of miRNA set D. The lncRNA-miRNA-mRNA network was constructed by assembling all the identified co-expression competing triplets, and was visualized using Cytoscape software (v3.6.0).^3^

### Functional enrichment analysis

GO functional and KEGG pathway analysis of miRNA, lncRNA, and circRNA target genes was conducted using DAVID and KOBAS software (22, 23). Go terms and KEGG pathways with a *p*-value <0.05 were considered to be significantly enriched.

### Validation of RNA-seq results by real-time quantitative PCR

Real-time quantitative PCR (RT-qPCR) was performed to validate the expression levels of DE circRNAs, DE lncRNAs, and DE miRNAs. Four lncRNAs, four miRNAs, and four circRNAs associated with significantly differentially expressed genes in the ovary were randomly selected for the RT-qPCR validation tests. The methods of RNA reverse transcription and real-time fluorescence quantitative PCR were conducted as per previously published protocols (24). The relevant primers were designed using Primer Premier. As stated earlier, U6 should be referred to as the steward gene sequence of miRNA (23). The methods of RNA reverse transcription and real-time fluorescence quantitative PCR were conducted as per previously published protocols (24). While GAPDH was used as the housekeeping gene for lncRNA and circRNA. The relative miRNA and mRNA expression were calculated using the 2^−∆∆Ct^ method (25), and data were expressed as mean ± standard deviation of the mean. The data was displayed as mean ± standard deviation, with variations analyzed via the independent-sample *t*-test, via SPSS 20.0 (SPSS Inc., United States).

## Results

### Effect of broodiness behavior on ovarian phenotype

**Figure 1:**
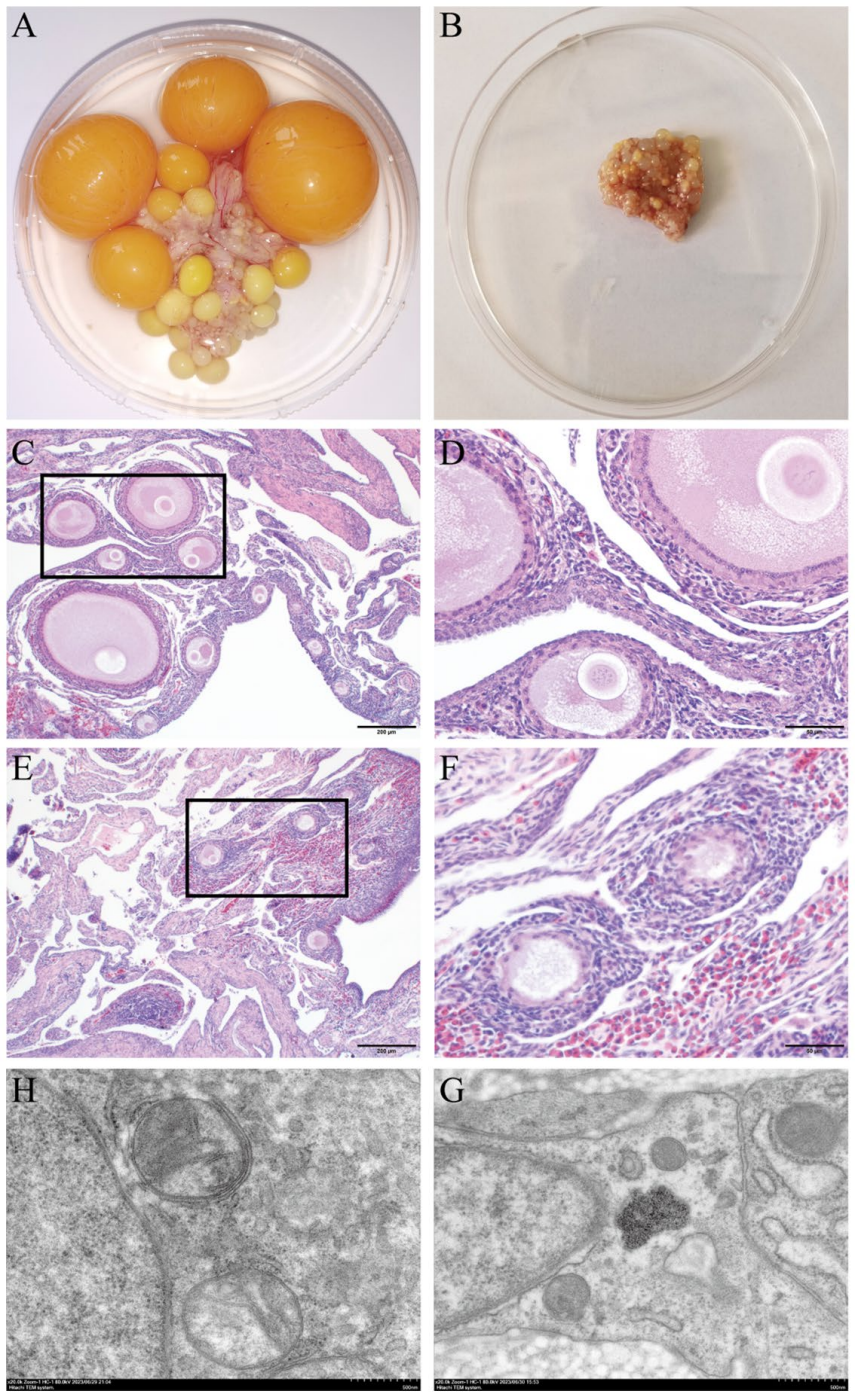
Anatomical morphological observation of ovarian tissue in poultry was conducted by examining two hematoxylin and eosin stained sections. **(A)** Normal ovarian tissue that continuously lays eggs. **(B)** Atrophy of ovaries for 30 days of broodiness. **(C)** Histological characteristics in egg-laying hens (staining at 200 μm). **(D)** Histological characteristics in egg-laying hens (staining at 50 μm). **(E)** Histological characteristics in broody hens (staining at 200 μm). **(F)** Histological characteristics in broody hens (staining at 50 μm). **(G)** The mitochondrial ultrastructure of the chicken egg-laying ovaries were analyzed by TEM. **(H)** The mitochondrial ultrastructure of the chicken broody ovaries were analyzed by TEM.

The ovarian volume diminished as the laying stage progressed to the broodiness stage (Figures 1A,B). During the laying stage, the ovaries contain varying developmental and atretic follicles. However, follicle development stops, and different levels of atretic follicles are observed when approaching the broodiness stage. Noteworthy, the egg-laying hens had a higher ovary weight and volume and a heftier stroma weight, than the broody chicken (Table 1). Moreover, the broody chicken did not have large yellow follicles. The hen only had a few yellow follicles, with the majority being white.

**Table 1:** A comparison of the ovarian morphological characteristics between egg-laying and broody hens (*n* = 6).

Tarits	EH	BC
Body weight (kg)	2.65 ± 0.12^*^	1.73 ± 0.14
Ovary weight (g)	49.62 ± 4.75^**^	2.73 ± 0.09
Ovary weight ratio	1.87 ± 0.18^**^	0.16 ± 0.02
Ovary volume (cm^3^)	48.32 ± 2.13^**^	4.58 ± 0.38
LYF (>10 mm, count)	5.25 ± 1.15	/
SYF (5–10 mm, count)	11.47 ± 0.62	/
WF (2–5 mm, count)	18.35 ± 4.97	18.03 ± 2.75
Stroma weight (g)	6.92 ± 1.15^**^	2.68 ± 0.07

EH, egg-laying hens; BC, broody chicken; WF, white follicles; LYF, large yellow follicle; SYF, small yellow follicles; ^*^p < 005 and ^**^p < 0.01.

The ovaries of chicken in the broodiness group had fewer follicles, a thicker granular layer, sparse cell density, and indistinct inner and outer layers compared to the ovaries of hens in the normal laying group (Figures 1C–F). Moreover, the ultrastructure of the ovary of chicken in the broodiness group revealed a lack of nucleus and a pattern of mitochondrial aggregation (Figures 1G,H).

### Effect of two different ovaries on miRNA

**Figure 2:**
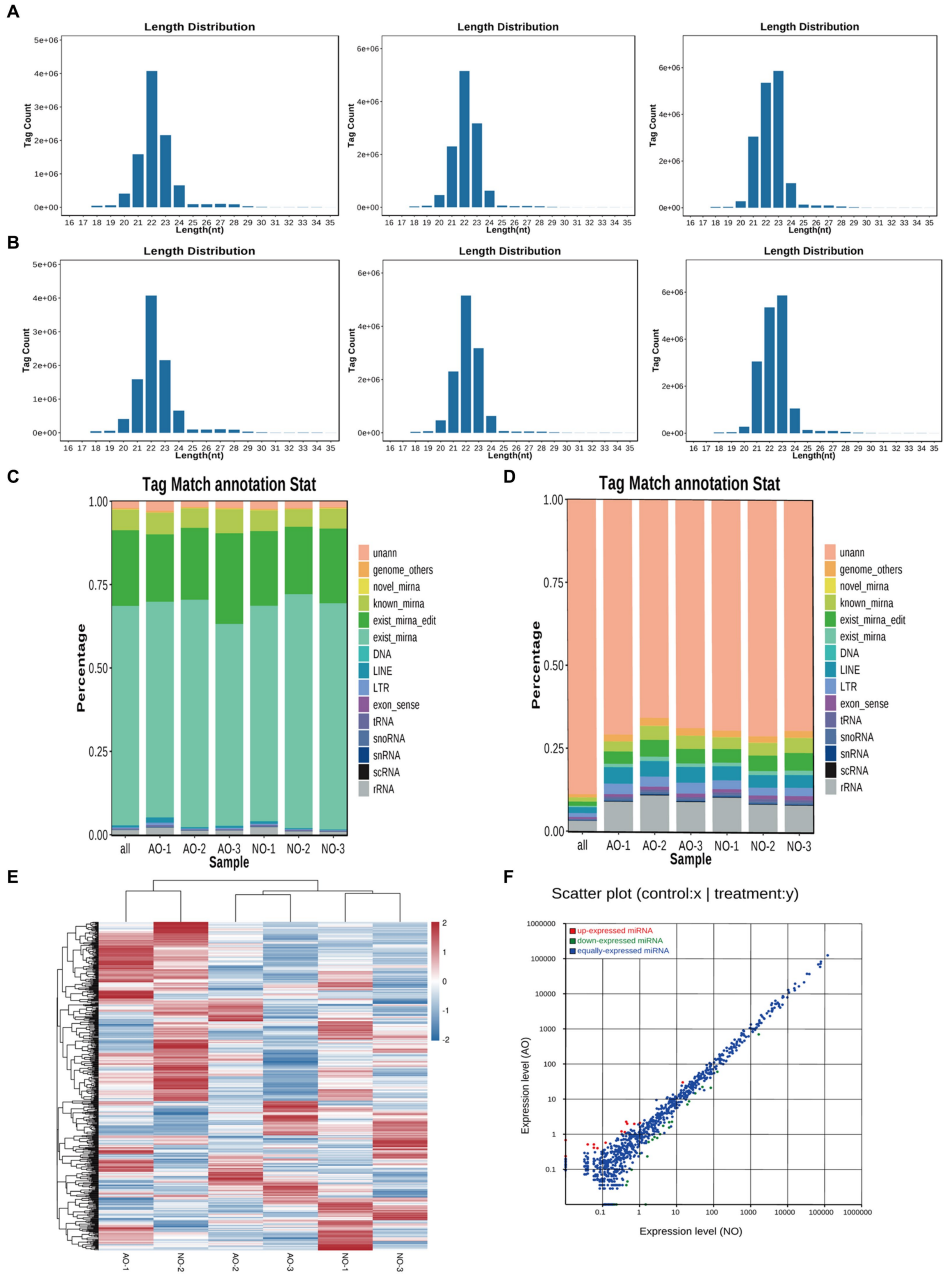
Overview of small RNA sequencing in the chicken ovary. **(A)** The length distribution of small RNA was AO1, AO2 and AO3, respectively. **(B)** The length distribution of small RNA was NO1, NO2 and NO3. **(C)** Statistical analysis of miRNA sequence abundance across various categories in each sample. **(D)** Statistical graph depicting the distribution of miRNA sequence species across various sample categories. exist_mirna (The miRNA of this species has been included in miRbase). known_miRNA (The identified miRNAs were compared with the known animal miRNAs in miRbase). novel_mirna (Combined with the reference sequence, the issuer structure was predicted and the miRNA was identified.) unann (The tags that cannot be annotated as any of the above molecules were recorded as unann). **(E)** Heatmap of differentially expressed miRNAs. **(F)** Compare the group NO –vs. -AO scatter plots.

**Figure 3:**
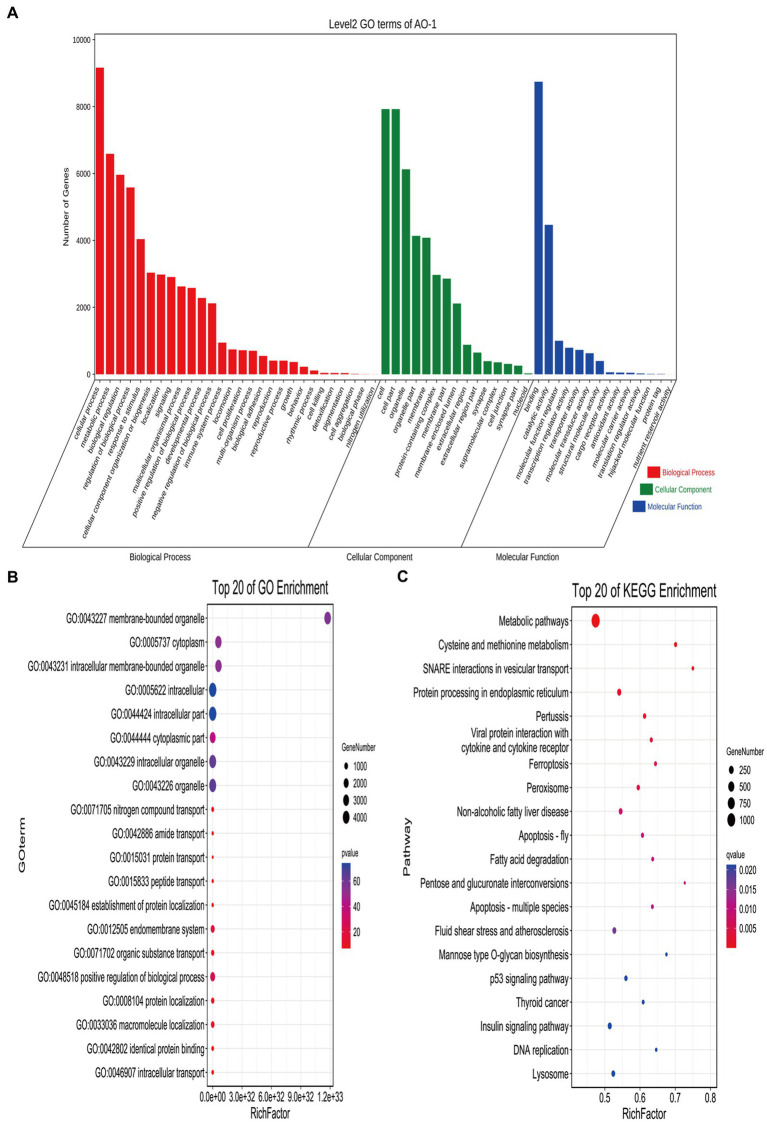
GO and KEGG analysis of DE miRNAs. **(A)** The GO enrichment classification histogram of DEmiRNA is divided into three levels: BP, MF, and CC. **(B)** Top 20 significantly changed GOs of DEmiRNAs in biological processes. **(C)** The top 20 pathways significantly associated with differentially expressed miRNA transcripts.

Transcriptome sequencing yielded 38,460,846 and 37,304,337 total raw reads from the broodiness and egg-laying groups, respectively. Subsequent filtering of the raw reads was done to acquire clean tags with more than 97% accuracy. As Figures 2A,B shown, 96.8% of miRNAs were between 20 and 24 nt in length (Supplementary material 1). In Figures 2C,D, plots are shown comparing the tag abundances of non-coding RNA in GenBank and Rfam for each sample. The proportion of existing miRNA is the highest. A miRNA expression heat map was subsequently generated for each sample to study the miRNA patterns in the samples (Figure 2E). As shown in Supplementary Figure S4, PCA principal component analysis showed intersample repeatability of AO (atrophic ovaries) and NO (normal ovaries). We also provide a violin diagram in Supplementary Figure S6, showing better correlation between samples. Moreover, 40 differential expression (DE) miRNAs were identified in comparing the broodiness and laying groups. A scatterplot analysis was performed based on the significantly different miRNAs to demonstrate the differences between the comparison groups (Figure 2F). GO and KEGG analysis of the 40 DE miRNA genes was then done to investigate the role of miRNA in the laying and broodiness groups. A total of 104 GO terms were enriched. Figures 3A,B shows the top 20 terms of biological process (BP), cellular component (CC), and molecular function (MF). GO enrichment analysis revealed that the DE miRNAs were mainly enriched in membrane-bounded organelle cytoplasm and some intracellular-related items. Additionally, the target genes were found to be involved in various metabolic pathways, including cysteine and methionine metabolism, SNARE interactions in vesicular transport, protein processing in endoplasmic reticulum, pertussis, viral protein interaction with cytokine and cytokine receptors, ferroptosis, and peroxisome pathways (Figure 3C).

### Effect of the two different ovaries on lncRNA

**Figure 4:**
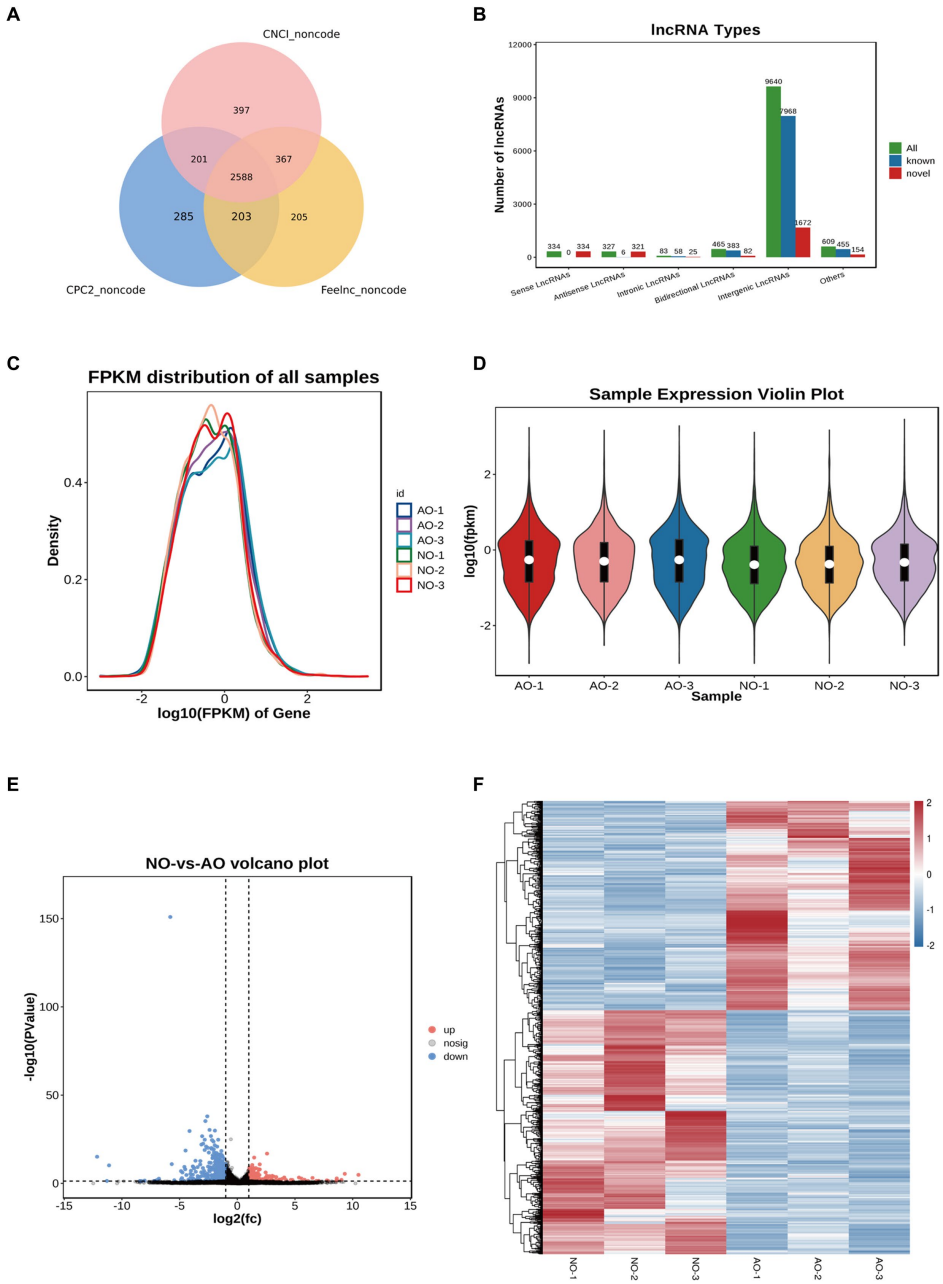
Overview of lncRNA sequencing in the chicken ovary. **(A)** CPC2, CNCI, and Feelnc were used to evaluate the encoding capabilities of all transcripts. The intersection of transcripts without coding potential is a reliable prediction result. **(B)** Total lncRNA type statistics. **(C)** lncRNA expression abundance distribution map. **(D)** Violin diagram of lncRNA expression. **(E)** Comparison of group NO-vs. -AO volcano maps. **(F)** Compare group NO-vs. -AO heat maps.

RNA-seq yielded 292,328,750 and 268,349,060 total raw reads from the broodiness and laying groups, respectively. Thus, to get high quality clean reads, reads were further filtered by fastp (26) (version 0.18.0). Filtering was then done to obtain a clean data set with more than 95% accuracy. The intersection of the three software results, which is depicted in the Venn diagram, was taken as the final lncRNA prediction result (Figure 4A). Noteworthy, 9,640 intergenic lncRNA, 465 bidirectional lncRNA, 83 intronic lncRNA, 327 antisense lncRNA and 334 sense overlapping lncRNA were identified based on the position of the new lncRNA relative to the protein-coding gene (Figure 4B). We also identified 2,588 new lncRNA for subsequent analysis. The expression of lncRNA and transcripts in different samples were visualized using expression distribution and violin maps (Figures 4C,D). By comparing the laying group and the broodiness group had 379 DE lncRNA (213 up-regulated and 166 down-regulated). The expression patterns are depicted in the volcanic maps and heat maps (Figures 4E,F). Our study revealed that ENSGALT00000105586 is the top DElncRNAs (Figure 4E).

GO analysis revealed that the lncRNA target genes were significantly enriched in processes related to extracellular activity, multicellular organismal processes, collagen fibril organization, and tissue development (Supplementary Figures S1A,B). The three most significant signaling pathways in the KEGG analysis of the lncRNA genes were ECM-receptor interaction, protein digestion and absorption, and focal adhesion (Supplementary Figure S1C).

### Effect of the two different ovaries on circRNA

**Figure 5:**
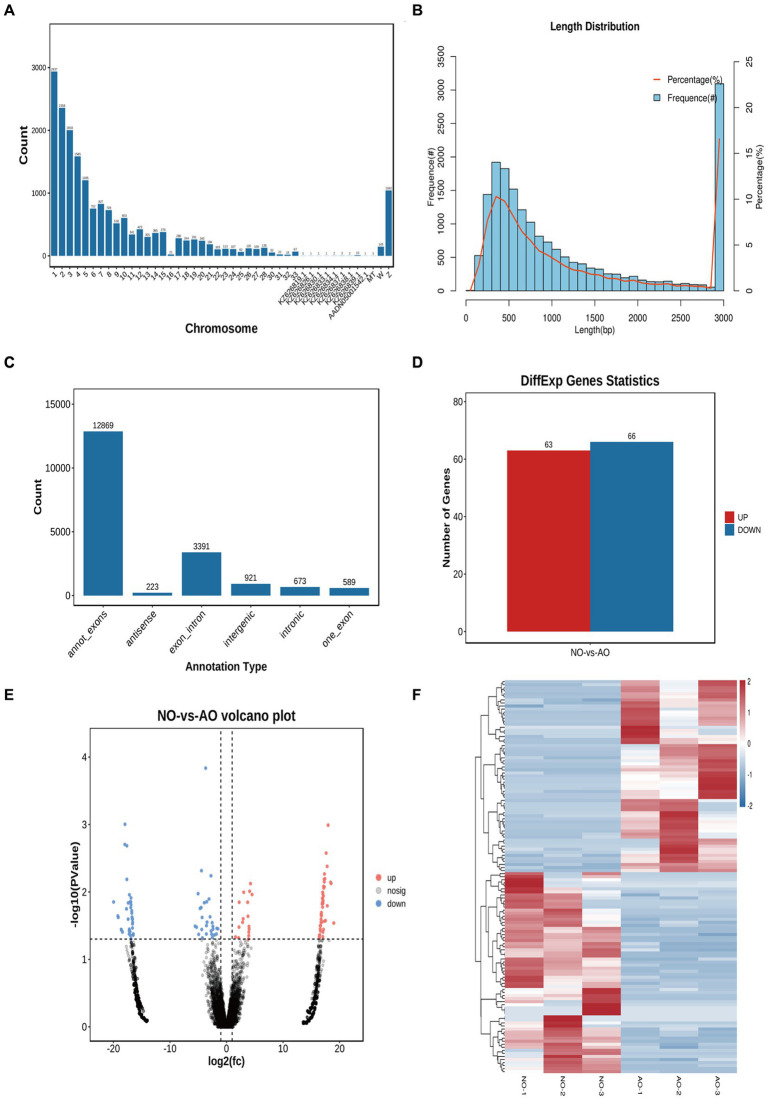
Overview of circRNA sequencing in the chicken ovary. **(A)** All the circular RNA chromosome statistics. **(B)** All the circular RNA length distribution statistics. **(C)** Statistical map of circular RNA type distribution. **(D)** CircRNA statistical map of differences. **(E)** Comparison of group NO-vs. -AO volcano maps. **(F)** Differential circRNA clustering heat map.

We obtained 55,838,523 clean reads from 6 samples after strict filtering (Supplementary material 3). A total of 18,666 circRNAs were subsequently identified from the clean reads in both the broodiness and laying groups (Supplementary Table S3). Notably, these circRNAs were distributed on almost all chromosomes, with the highest concentration on chromosome 1 (Figure 5A). A majority (83.82%) of circRNAs had a length of less than 3,000 bp, amongst which 30.55% were less than 500 bp and 47.42% were between 500–2,000 bp, with an average length of 3,156 bp (Figure 5B). The most abundant circRNA type was annot_exons (12,869; Figure 5C). A comparison between the expression levels of circRNA in the broodiness group and the laying group yielded 129 DE circRNAs (*p* < 0.05; Figure 5D). These 129 DE circRNAs were further analyzed using a volcanic map, we has been determined in our investigation that novel_circ_018025 stands out as a top DEcircRNA (Figure 5E). A heat map analysis of the differential circRNA expression patterns was also conducted (Figure 5F).

GO enrichment analysis of the DE circRNAs revealed their involvement in histone demethylase, phosphoprotein phosphatase, and metalloendopeptidase activities (Supplementary Figure S2B). KEGG enrichment analysis further revealed that DE circRNAs were linked to adherens junction, lysine degradation, cell adhesion molecules, thiamine metabolism, and PI3K-Ark signaling pathway (Supplementary Figure S2C).

### Construction of ceRNA networks associated with ovarian atrophy of the two different ovaries

**Figure 6:**
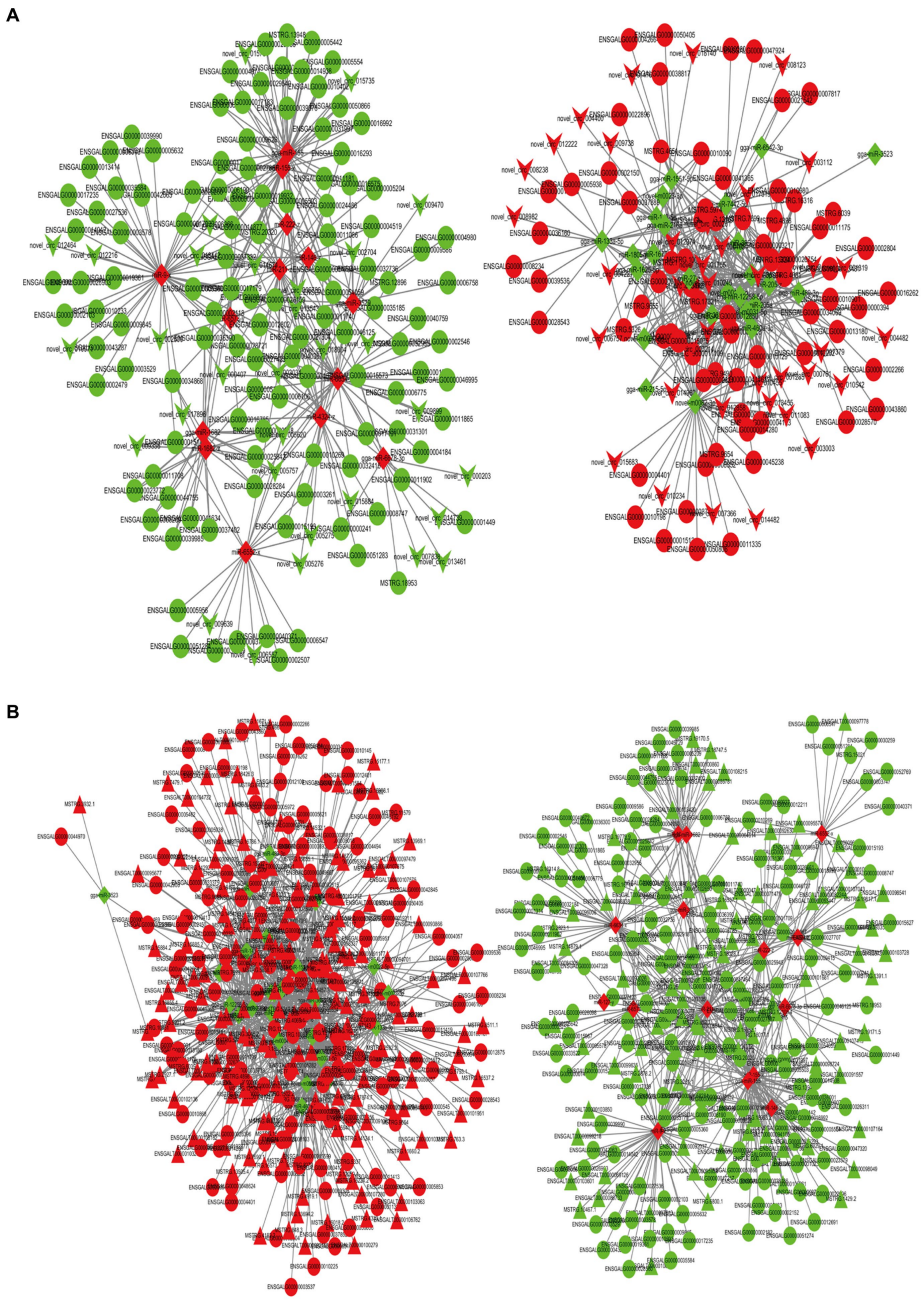
CeRNA network constructed by DEmRNAs, DElncRNAs, DEcircRNAs, and DEmiRNAs. **(A)** mRNA-miRNA-circRNA network. **(B)** mRNA-miRNA-lncRNA network. The shades of the colors indicate the upregulated (red) and downregulated (green) level. The characters of the figures correspond to different RNA species, round -mRNA, diamond-miRNA, v-circRNA, triangle-lncRNA.

An analysis of the differential expression of miRNAs in the ovarian tissue revealed 258 lncRNA-miRNA-mRNA and 70 circRNA-miRNA-mRNA interaction pairs, encompassing 291 lncRNAs, 79 circRNAs, 40 miRNAs, and 346 mRNAs (Figures 6A,B). GO and KEGG enrichment analyses (Supplementary Figures S5A,B) revealed that these transcriptional interactions were associated with ECM-receptor interaction, AGE-RAGE signaling pathway, focal adhesion, cytokine–cytokine receptor interaction, inflammatory mediator regulation of TRP channels, renin secretion, gap junction, insulin secretion, serotonergic synapse, and IL-17 signaling pathway. Through KEGG and GO analysis, we identified differentially expressed genes within relevant pathways. Furthermore, 25 genes potentially associated with broodiness were identified (Table 2).

**Table 2:** Twenty-five candidate genes associated with the regulation of broodiness and their binding pathways, and differentially expressed miRNA, lncRNA, and circRNA.

Differentially expressed genes	Pathway	Related differentially expressed miRNA	Related lncRNA	Related circRNA
COMP	PI3K-Akt signaling pathway	miR-211-z	ENSGALT00000102650	
FN1	Pathways in cancer	miR-9-x	ENSGALT00000069126, ENSGALT00000097813, ENSGALT00000099218, ENSGALT00000103601, MSTRG.10467.1	novel_circ_010071
ITGA8	PI3K-Akt signaling pathway	miR-4324-z	ENSGALT00000103728, ENSGALT00000105373, MSTRG.19817.1	novel_circ_015884
THBS1	PI3K-Akt signaling pathway	gga-miR-155, miR-155-x, miR-211-z	ENSGLT0000055899, ENSGALT00000093080, ENSGALT00000097500, ENSGALT00000098724, ENSGALT00000100307, MSTRG.12108.4, MSTRG.18017.1, MSTRG.2399.1, MSTRG.2955.5, MSTRG.3306.4, MSTRG.8753.4	novel_circ_013543, novel_circ_013544, novel_circ_013545, novel_circ_014674
TNC	PI3K-Akt signaling pathway	miR-9-x	ENSGALT00000069126, ENSGALT00000086733, ENSGALT00000097813, ENSGALT00000099218, MSTRG.10467.1	novel_circ_010071
IL8	Pathways in cancer	miR-6573-y	ENSGALT00000095578	
CR1	Tuberculosis	gga-miR-143-5p	MSTRG.12254.4	
PF4	Pathways in cancer	novel-m0022-5p, novel-m0023-3p	ENSGALT00000094510, ENSGALT00000097728, ENSGALT00000098801, ENSGALT00000105534, ENSGALT00000107766, MSTRG.16216.1, MSTRG.2592.2, MSTRG.5783.5	
MAPK10	Pathways in cancer	gga-miR-1677-3p, gga-miR-489-3p, novel-m0012-3p, gga-miR-133a-5p, gga-miR-205a, gga-miR-365-3p, miR-205-z, novel-m0082-3p, gga-miR-1625-5p, novel-m0031-5p	ENSGALT00000092815, ENSGALT00000097073, MSTRG.13979.1, MSTRG.4262.1	
AGT	Pathways in cancer	gga-miR-1551-5p, gga-miR-365-3p	ENSGALT00000091177, ENSGALT00000102866, MSTRG.16427.1, MSTRG.3924.2, MSTRG.5783.5	
COL3A1	Amoebiasis	miR-9-x	ENSGALT00000069126, ENSGALT00000086733, ENSGALT00000097813, ENSGALT00000099218, ENSGALT00000103601, ENSGALT00000106808, MSTRG.10467.1	novel_circ_010071
SMAD2Z	Pathways in cancer	gga-miR-155, miR-155-x	ENSGALT00000092121, ENSGALT00000097500, MSTRG.18017.1, MSTRG.2399.1, MSTRG.6929.1	novel_circ_006786, novel_circ_014674
MYLK	Calcium signaling pathway	gga-miR-1682, miR-1682-z	ENSGALT00000034451, ENSGALT00000064614, ENSGALT00000098372, ENSGALT00000108215, MSTRG.18028.1, MSTRG.18747.5, MSTRG.19170.5, MSTRG.3306.4	novel_circ_014674
PDGFD	PI3K-Akt signaling pathway	gga-miR-1682, miR-1682-z, gga-miR-155, miR-155-x, miR-222-z, miR-9-x	ENSGALT00000055899, ENSGALT00000095958, ENSGALT00000097500, ENSGALT00000100860, MSTRG.18017.1, MSTRG.19170.5, MSTRG.3306.4	novel_circ_009336, novel_circ_013543, novel_circ_014674
F2	Pathways in cancer	novel-m0031-5p, novel-m0082-3p	MSTRG.14124.1, MSTRG.15941.1, MSTRG.4656.1	novel_circ_011083
KCNMB1	cGMP-PKG signaling pathway	miR-211-z, miR-9-x, gga-miR-1682, miR-1682-z, miR-155-x	ENSGALT00000029552, ENSGALT00000034451, ENSGALT00000095958, ENSGALT00000100307, ENSGALT00000108215, MSTRG.18028.1, MSTRG.18747.5, MSTRG.19170.5, MSTRG.3306.4 MSTRG.852.1 MSTRG.8753.4	novel_circ_009336, novel_circ_014674, novel_circ_018442
KCNMA1	cGMP-PKG signaling pathway	gga-miR-3529	ENSGALT00000098838	novel_circ_009470
ACTA2	Apelin signaling pathway	miR-9-x	ENSGALT00000069126, ENSGALT00000086733, ENSGALT00000097813, ENSGALT00000099218, ENSGALT00000103601, ENSGALT00000106808, MSTRG.10467.1	novel_circ_010071
GNAQ	Pathways in cancer	miR-6552-x, miR-9-x		novel_circ_003034, novel_circ_017896
CXCR5	Cytokine-cytokine receptor interaction	gga-miR-143-5p, gga-miR-7442-5p	MSTRG.4518.5	
CCR7	Cytokine-cytokine receptor interaction	gga-miR-12258-5p, novel-m0012-3p	MSTRG.5730.1, MSTRG.9716.2	
CAMK2A	Pathways in cancer	gga-miR-143-5p	ENSGALT00000095677, MSTRG.12254.4	
HTR2C	Neuroactive ligand-receptor interaction	novel-m0031-5p	ENSGALT00000092468	
CYP2J2	Metabolic pathways	novel-m0012-3p	ENSGALT00000091079, ENSGALT00000103356	
MMP9	Pathways in cancer	gga-miR-155, miR-155-x, miR-6573-y	ENSGALT00000055899, ENSGALT00000097500, ENSGALT00000098724, ENSGALT00000100307, MSTRG.12108.4, MSTRG.18017.1, MSTRG.2399.1, MSTRG.2955.5, MSTRG.3306.4, MSTRG.8753.4	novel_circ_003034

**Figure 7:**
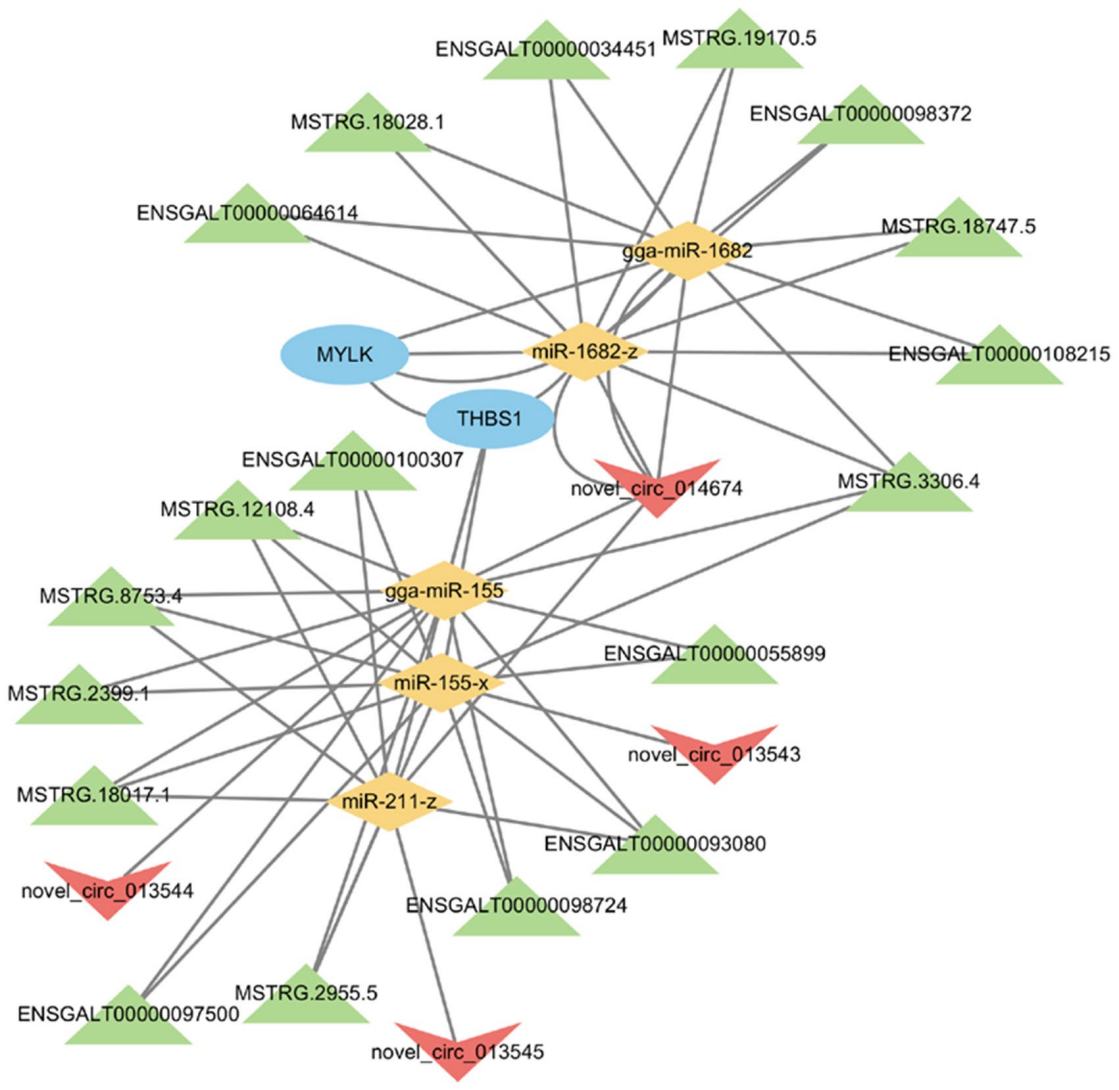
lncRNA/circRNA-miRNA-mRNA co-regulatory network. The characters of the figures correspond to different RNA species, round -mRNA, diamond -miRNA, to v-circRNA, triangle -lncRNA.

A lncRNA/circRNA-miRNA-mRNA regulatory network was subsequently created by constructing ceRNA networks of candidate genes (Figure 7). In the network, miRNAs were at its center and was associated with 4 circRNAs, 18 lncRNAs, 5 miRNAs, and 2 mRNAs. The ceRNA network analysis identified *MYLK* and *THBS1* genes as potential targets of 2 and 3 differentially expressed miRNAs, and 8 and 10 lncRNAs, respectively. Moreover, four circRNAs had a regulatory effect on *MYLK* and *THBS1* genes. The novel_circ_014674 and MSTRG.3306.4 potentially regulated *MYLK* and *THBS1* genes via different miRNAs, which was consistent with the ceRNA regulatory hypothesis (27).

### RT-qPCR validation of DE circRNAs, DE lncRNAs, and DE miRNAs

**Figure 8:**
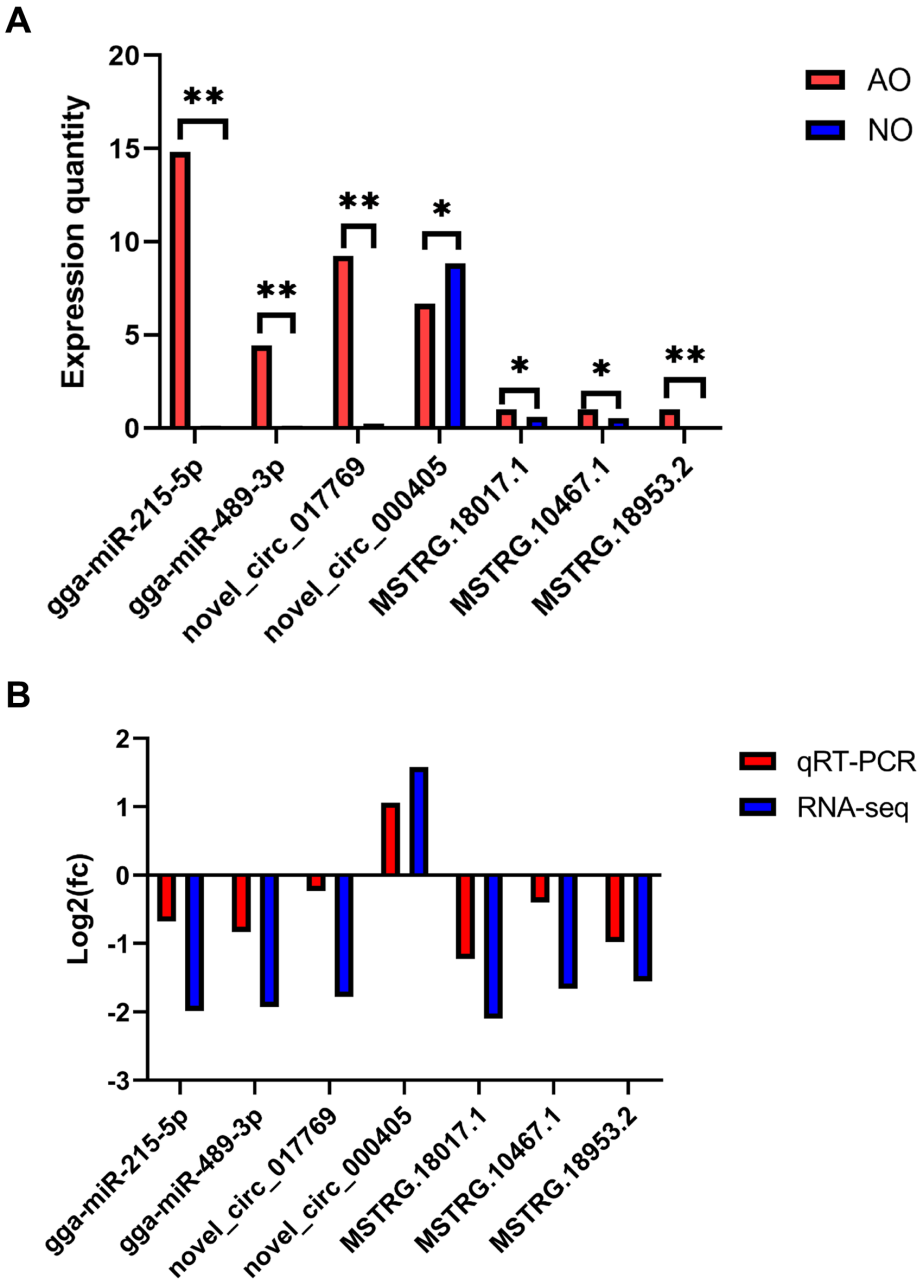
Validation of RNA-seq data using real time quantitative PCR (RT-qPCR).

Seven transcripts were randomly selected from DE circRNA, DE lncRNA, and DE miRNA and subjected to RT-qPCR to verify the accuracy of the RNA sequencing data. Subsequent RT-qPCR validation demonstrated extremely significant differences (*p* < 0.01) in gga-miR-215-5p, gga-miR-489-3p, novel_circ_017769, and MSTRG.18953.2. Additionally, novel_circ_000405, MSTRG.18017.1, and MSTRG.10467.1 exhibited significant differences (*p* < 0.05), as depicted in Figure 8A. The log2FC values of DE miRNA, DE lncRNA, and DE circRNA obtained from the RT-qPCR results revealed that the seven genes exhibited a consistent expression trend with those of RNA-Seq data, thus confirming the accuracy of the sequencing results in Figure 8B.

## Discussion

Poultry broodiness is a complex characteristic with low heritability, it is a result of a combination of genetic influences, the endocrine system, and environmental factors (28). Broody behavior frequently results in follicular atresia and ovarian atrophy (29). In addition, the adoption of artificial incubation technology has eliminated the need for broody behavior in chicken production. Consequently, identifying potential targets that impact broody behavior serves as a method to enhance egg production in native chicken populations. The ovaries and fallopian tubes of broody chickens undergo degeneration, which impedes the growth of follicles and reduces the number of granulosa and thecal cells within them, thus hindering egg production (4, 30). During the brooding stage, Magang geese have very few large yellow follicles and small yellow follicles, yet the amount of large white follicles remains unchanged (31). In this study, histological examination revealed that the ovaries of broody chickens had atrophied, with atretic follicles, which is consistent with the results of other studies (32, 33). Transmission electron microscope (TEM) images of the ovarian ultrastructure suggested that the ovarian granulosa cells of polycystic ovarian syndrome (PCOS) model rats exhibited an accumulation of mitochondria, destruction of crests, and breaking up as opposed to those of normal rats (34). Granulosa cells with dysfunctional mitochondria can lead to a decrease in steroid production and oocyte maturation rate, resulting in reduced fertility. Conversely, functional mitochondria increase the steroid production of granulosa cells (35).

Non-coding RNAs are important in the biological processes of living organisms. The ceRNA hypothesis enables the exploration of potential regulatory mechanisms by combining multiple RNA information (36). In this study, we identified 40 differentially expressed miRNAs (DEmiRNAs), 379 differentially expressed long non-coding RNAs (DElncRNAs), and 129 differentially expressed circular RNAs (DEcircRNAs) in the ovaries of laying chickens and those with broodiness. Subsequent enrichment analysis enabled the construction of a hierarchical ceRNA network associated with ovarian development. The network identified miRNAs, such as miR-9-x, miR-4324-z, miR-155-x, gga-miR-143-5p, and miR-211-z, miR-155, miR-9, and miR-4324 are involved in the development of the ovary. miR-155 has an essential effect on the glycolysis of granule cells in PCOS sufferers, thereby controlling follicular dysplasia (37). miR-9 is highly expressed in the follicular fluid of PCOS patients, and its interaction with the vitamin D receptor modulates the proliferation and apoptosis of ovarian granulosa cells, thereby regulating follicle growth (38–40). Moreover, miR-9 increases during the ovarian development of tilapia, thereby affecting sex determination and differentiation by regulating dmrt1 expression (41). Notably, there are differences in the development process of miR-9 in different regions of the chick’s brain (42). miR-4324 is associated with cell proliferation and migration. Its expression is thus significantly reduced in ovarian cancer tissues. miR-4324 can be targeted and combined with *FEN1* to impede cell proliferation in ovarian cancer (43, 44). Recent studies postulate that miR-143 has a regulatory effect on glucose metabolism and mitochondrial function in the placenta (45). Yang et al. (46) reported that miR-143-5p combines with *CRIM1* to regulate endometrial receptivity in goats. In the same line, studies postulate that the regulatory axis of lncRNA PROX1-AS1, miR-211-5p, and caspase-9 is connected to preeclampsia and affects the presence of trophoblast cells (47).

Herein, miRNA, lncRNA, and circRNA source genes were significantly enriched in the ECM-receptor interaction pathway. ECM receptors are associated with the ripening of follicles and the reduction of the ovaries (34, 48). Research has demonstrated that extracellular matrix (ECM) components furnish tissue-specific frameworks that impact key cellular processes, including proliferation, differentiation, and apoptosis. The extensive alterations in ECM throughout follicular development necessitate a sequence of proteolytic events crucial for the regular progression, function, regression, and degeneration of follicles (49). Recent studies have indicated that matrix proteins play a significant role in regulating ECM turnover during follicular development or atresia. Apart from specifically degrading ECM components and activating other matrix metalloproteinases, these proteins also influence various signaling pathways by releasing numerous signaling proteins. This regulation impacts cell biology during both normal physiological processes and pathological conditions, involving growth factors, cytokines, cadherin E, Fas ligands, and tumor necrosis factors (50). Simultaneously, we observed potential regulatory interactions between miR-9-x and the genes *FN1, TNC, COL3A1, PDGFD, KCNMB1, ACTA2,* and *GNAQ* through the ceRNA network. Additionally, previous studies have indicated that *FN1* is a principal constituent of the extracellular matrix (ECM) (51). Furthermore, an examination of high and low egg production in white Muscovy ducks identified potential candidate genes in both hypothalamic and ovarian tissue for the high-yield mechanism in this breed. Notably, the *FN1* gene displayed significant expression levels in ovarian tissue and is implicated in this mechanism (52). Focal adhesion was also a significant pathway in the ceRNA network in this study. Previous studies have demonstrated its involvement in cell proliferation (53). Moreover, studies postulate that focal adhesion is a factor in the expression of the IGF-I gene and results in a difference in the gene’s expression in Muscovy ducks. IGF-I is responsible for controlling the expression of lncRNA through the focal adhesion pathway, thereby promoting cell proliferation and regulating the reproduction of Muscovy ducks (54). ECM-receptor interaction and focal adhesion are involved in the early development of geese and ducks ovaries (48). lncRNAs regulate the functioning of chicken ovaries through their interaction with ECM-receptor and focal adhesion pathways (55). In this study, focal adhesion was found to be an enrichment pathway associated with higher and lesser egg production, which is in line with other research findings (56). Through additional analysis of the ceRNA network, we have pinpointed two crucial genes: *THBS1* and *MYLK*. However, there has been limited research on poultry involving *THBS1* and *MYLK*. Increased *THBS1* expression in rat follicles has been demonstrated to notably diminish angiogenesis while enhancing follicular atresia (57). In another study on ovarian cancer, it was observed that the inhibition of circRNA_MYLK led to a decrease in the proliferation of ovarian cancer cells (58). We hypothesized that ncRNAs modulate the broodiness behavior of poultry by regulating *THBS1* and *MYLK* gene expression. The findings of this study revealed the existence of a regulatory connection between ncRNAs and their competing mRNAs, which provides a better understanding of the ceRNA function and processes in chicken ovarian development.

## Conclusion

This study reveals 40 DE miRNAs, 379 DE lncRNAs, and 129 DE circRNAs from the ovaries of laying chickens and hens with broodiness. *THBS1* and *MYLK* are candidate genes in the regulation of broody behavior, potentially influenced by miR-155-x, miR-211-z, miR-1682-z, gga-miR-155, and gga-miR-1682. Additionally, they may be subject to competitive binding by novo-el_circ_014674 and MSTRG.3306.4. The GO and KEGG enrichment analyses revealed a connection between the ECM-receptor interaction pathway and focal adhesion in the regulation of broody behavior, offering a novel avenue for investigating the regulatory network of Chengkou mountain chicken’s broody behavior further.

## Data availability statement

The raw data has been furnished to the National Center for Biotechnology Information (NCBI) Sequence Read Archive (SRA) database, assigned the accession number PRJNA1074948.

## Ethics statement

The animal study was approved by Experimental Animal Ethics Review Committee of Southwest University. The study was conducted in accordance with the local legislation and institutional requirements.

## Author contributions

HX: Data curation, Software, Validation, Writing – original draft. WL: Formal analysis, Validation, Writing – review & editing. LW: Formal analysis, Writing – review & editing. XW: Data curation, Writing – review & editing. BT: Conceptualization, Formal analysis, Supervision, Writing – review & editing. ZC: Conceptualization, Software, Writing – review & editing. LL: Funding acquisition, Project administration, Resources, Writing – original draft, Writing – review & editing.

## Footnotes

^1^ http://cpc.cbi.pku.edu.cn/

^2^ https://github.com/tderrien/FEELn

^3^ http://www.cytoscape.org/
