# Supplementary material for: Whole transcriptome analysis revealed the regulatory network and related pathways of non-coding RNA regulating ovarian atrophy in broody hens
Source: Front Vet Sci. 2024 May 29;11:1399776. doi: 10.3389/fvets.2024.1399776 (PMC11168117; doi:10.3389/fvets.2024.1399776)
Supplement: Supplementary file 2 [file Table_2.docx]

**Table 1** Statistics of sample data filtering and disconnecting.

| id | clean_reads | high_quality | 3'adapter_null | insert_null | 5'adapter_contaminants | polyA | clean_tags |
| --- | --- | --- | --- | --- | --- | --- | --- |
| AO-1 | 9733511 (100%) | 9595004 (98.5770%) | 5592 (0.0583%) | 10677 (0.1113%) | 4271 (0.0445%) | 68 (0.0007%) | 9444074 (97.0264%) |
| AO-2 | 12337758 (100%) | 12176215 (98.6907%) | 28976 (0.2380%) | 9912 (0.0814%) | 2334 (0.0192%) | 113 (0.0009%) | 12054974 (97.7080%) |
| AO-3 | 16389577 (100%) | 16202911 (98.8611%) | 11356 (0.0701%) | 21493 (0.1326%) | 3168 (0.0196%) | 93 (0.0006%) | 16086086 (98.1483%) |
| NO-1 | 11155346 (100%) | 10990199 (98.5196%) | 7878 (0.0717%) | 17664 (0.1607%) | 3539 (0.0322%) | 94 (0.0009%) | 10850010 (97.2629%) |
| NO-2 | 12396623 (100%) | 12191567 (98.3459%) | 41996 (0.3445%) | 13919 (0.1142%) | 3509 (0.0288%) | 168 (0.0014%) | 12029170 (97.0359%) |
| NO-3 | 13752368 (100%) | 13554864 (98.5639%) | 18745 (0.1383%) | 13139 (0.0969%) | 2597 (0.0192%) | 64 (0.0005%) | 13457925 (97.8590%) |

**Table 2** LncRNA data filtering statistics table.

| Sample | RawDatas | CleanData(%) | Adapter(%) | LowQuality(%) | polyA(%) | N(%) |
| --- | --- | --- | --- | --- | --- | --- |
| AO-1 | 99628398 | 99299106 (99.67%) | 39536 (0.04%) | 289738 (0.29%) | 0 (0.00%) | 18 (0.00%) |
| AO-2 | 99033040 | 98653412 (99.62%) | 44460 (0.04%) | 334996 (0.34%) | 0 (0.00%) | 172 (0.00%) |
| AO-3 | 93667312 | 93291062 (99.60%) | 39038 (0.04%) | 337074 (0.36%) | 0 (0.00%) | 138 (0.00%) |
| NO-1 | 96643248 | 96207458 (99.55%) | 42490 (0.04%) | 393154 (0.41%) | 0 (0.00%) | 146 (0.00%) |
| NO-2 | 83036242 | 82678628 (99.57%) | 33154 (0.04%) | 324330 (0.39%) | 0 (0.00%) | 130 (0.00%) |
| NO-3 | 88669570 | 88255564 (99.53%) | 45618 (0.05%) | 368378 (0.42%) | 0 (0.00%) | 10 (0.00%) |

**Table 3** CircRNA data filtering statistics table.

| Sample | RawDatas | CleanData(%) | Adapter(%) | LowQuality(%) | polyA(%) | N(%) |
| --- | --- | --- | --- | --- | --- | --- |
| AO-1 | 99628398 | 99299106 (99.67%) | 39536 (0.04%) | 289738 (0.29%) | 0 (0.00%) | 18 (0.00%) |
| AO-2 | 99033040 | 98653412 (99.62%) | 44460 (0.04%) | 334996 (0.34%) | 0 (0.00%) | 172 (0.00%) |
| AO-3 | 93667312 | 93291062 (99.60%) | 39038 (0.04%) | 337074 (0.36%) | 0 (0.00%) | 138 (0.00%) |
| NO-1 | 96643248 | 96207458 (99.55%) | 42490 (0.04%) | 393154 (0.41%) | 0 (0.00%) | 146 (0.00%) |
| NO-2 | 83036242 | 82678628 (99.57%) | 33154 (0.04%) | 324330 (0.39%) | 0 (0.00%) | 130 (0.00%) |
| NO-3 | 88669570 | 88255564 (99.53%) | 45618 (0.05%) | 368378 (0.42%) | 0 (0.00%) | 10 (0.00%) |

**Table 4** The primers for the RT-qPCR amplification

| Genes | Reverse transcription primer | Forward primer(5’-3’) |
| --- | --- | --- |
| gga-miR-215-5p | GTCGTATCCAGTGCAGGGTCCGAGGTATTCGCACTGGATACGACTATTGG | F: CGCGCCTGTCATTTCTATAGG |
|  |  | R: AGTGCAGGGTCCGAGGTATT |
| gga-miR-489-3p | GTCGTATCCAGTGCAGGGTCCGAGGTATTCGCACTGGATACGACGCGCCG | F: CGCGCGAGGACACAAGA |
|  |  | R: AGTGCAGGGTCCGAGGTATT |
| U6 | CCATATTAGAAGCCCCTTTTTGT | F: TCGCTTCGGCAGCACATA |
|  |  | R: AATATGGAACGCTTCACGAA |
| novel_circ_017769 | F: TCCATTCTCTCCCACCAAAGTG | R: CTTCATCTTCTGTAGCAATAACTCC |
| novel_circ_000405 | F: GCAGACTTATTTCAGGCATCGC | R: GTCCCAGCAACCAAACAGGA |
| MSTRG.18017.1 | F: ACAAAGCAGCAGCCTCAACATC | R: AGAGATAGCAGGTAGTCCAGCATTC |
| MSTRG.10467.1 | F: GGGAAGGGAAAGCAAAGCACAG | R: GATGGATGGATGAATGGATGGATGG |
| MSTRG.18953.2 | F: CCTTGGTGGCCGCCCTATG | R: ACCGTGAACTCCTGCTCCTTG |
| GAPDH | F: CAGAACATCATCCCAGCGT | R: CAGGTCAGGTCAACAACAG |
| 18SRNA | F: TGCATGTCTAAGTACACACGG | R: AGGTCGGCGCTCGTCGGCATG |

Note: F: forward primer, R: reverse primer.

**Table 5** Nutritional composition table for feed.

| Diet composition | content（%） | Nutrient level | content（%） |
| --- | --- | --- | --- |
| Corn | 66.00 | Metabolizable energy（MJ/kg） | 11.42 |
| Soybean meal | 26.00 | Crude protein | 16.00 |
| Stone powder | 7.00 | Calcium | 3.50 |
| Premix | 1.00 | Total phosphorus | 0.60 |
|  |  | Available phosphorus | 0.43 |
|  |  | Lysine | 0.80 |
