# Supplementary material for: Whole transcriptome analysis revealed the regulatory network and related pathways of non-coding RNA regulating ovarian atrophy in broody hens
Source: Front Vet Sci. 2024 May 29;11:1399776. doi: 10.3389/fvets.2024.1399776 (PMC11168117; doi:10.3389/fvets.2024.1399776)

Figure 1 GO and KEGG analysis of DElncRNAs. (A) The GO enrichment classification histogram of lncRNA target genes is divided into three levels: BP, MF, and CC**.** (B) Top 20 significantly changed GOs of DElncRNAs in biological processes**.** (C) The top 20 pathways significantly associated with differentially expressed lncRNA transcripts.

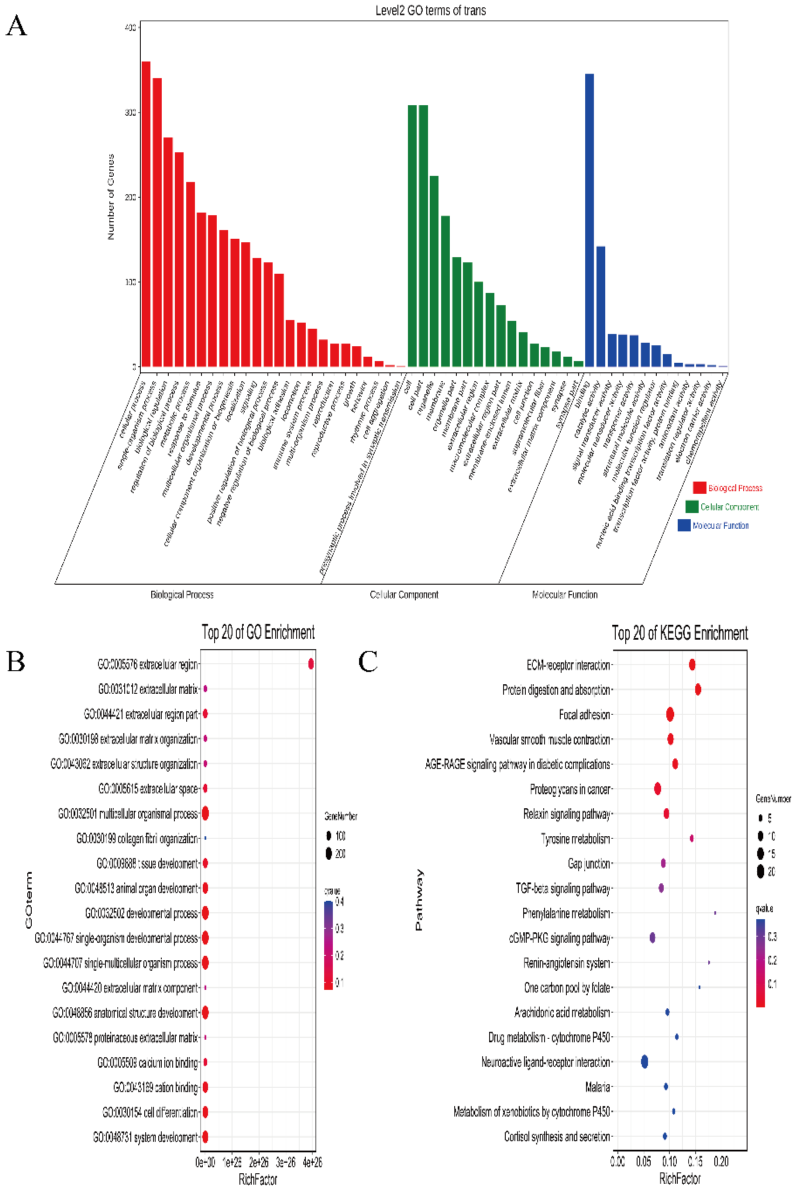

Figure 2 GO and KEGG analysis of DE circRNAs. (A) The GO enrichment classification histogram of circRNA target genes is divided into three levels: BP, MF, and CC (B) Top 20 significantly changed GOs of DEcircRNAs in biological processes**.** (C) The top 20 pathways significantly associated with differentially expressed circRNA transcripts

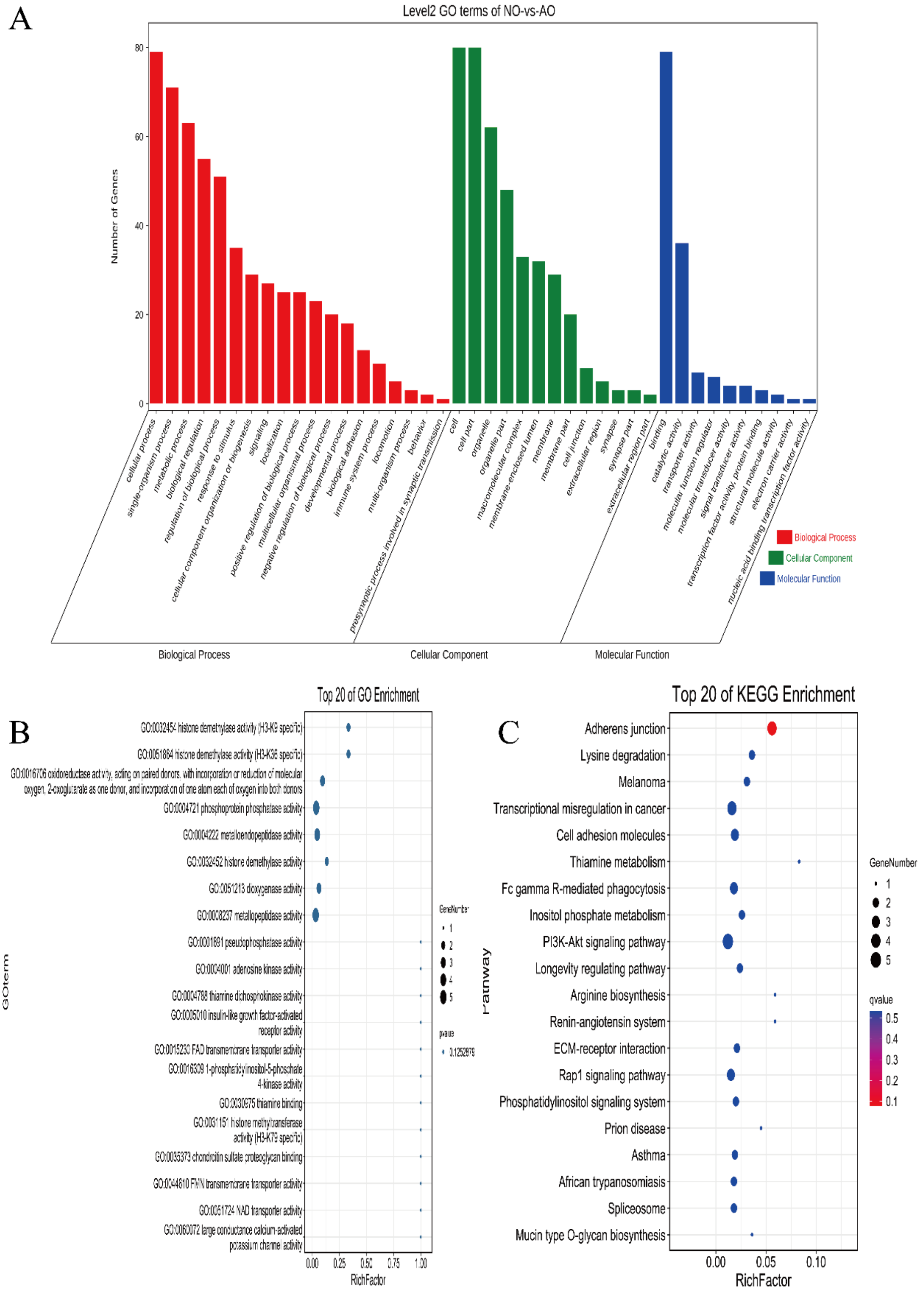
A
Level2 GO terms of NO-vs-AO
Number of Genes
0
20
40
60
80
cellular process
single-organism process
metabolic process
biological regulation
regulation of biological process
response to stimulus
cellular component organization or biogenesis
signaling
localization
positive regulation of biological process
multicellular organismal process
negative regulation of biological process
developmental process
biological adhesion
immune system process
locomotion
multi-organism process
behavior
presynaptic process involved in synaptic transmission
cell
cell part
organelle
organelle part
macromolecular complex
membrane-enclosed lumen
membrane
membrane part
cell junction
extracellular region
synapse
synapse part
extracellular region part
binding
catalytic activity
transporter activity
molecular function regulator
molecular transducer activity
signal transducer activity
transcription factor activity, protein binding
structural molecule activity
electron carrier activity
nucleic acid binding transcription factor activity
Biological Process
Cellular Component
Molecular Function
B
Top 20 of GO Enrichment
GOterm
GO:0032454 histone demethylase activity (H3-K9 specific)
GO:0051864 histone demethylase activity (H3-K36 specific)
GO:0016706 oxidoreductase activity, acting on paired donors, with incorporation or reduction of molecular oxygen, 2-oxoglutarate as one donor, and incorporation of one atom each of oxygen into both donors
GO:0004721 phosphoprotein phosphatase activity
GO:0004222 metalloendopeptidase activity
GO:0032452 histone demethylase activity
GO:0051213 dioxygenase activity
GO:0008237 metallopeptidase activity
GO:0001891 pseudophosphatase activity
GO:0004001 adenosine kinase activity
GO:0004788 thiamine diphosphokinase activity
GO:0005010 insulin-like growth factor-activated receptor activity
GO:0015230 FAD transmembrane transporter activity
GO:0016309 1-phosphatidylinositol-5-phosphate 4-kinase activity
GO:0030975 thiamine binding
GO:0031151 histone methyltransferase activity (H3-K79 specific)
GO:0035373 chondroitin sulfate proteoglycan binding
GO:0044610 FMN transmembrane transporter activity
GO:0051724 NAD transporter activity
GO:0060072 large conductance calcium-activated potassium channel activity
0.00
0.25
0.50
0.75
1.00
RichFactor
GeneNumber
1
2
3
4
5
pvalue
0.1252879
C
Top 20 of KEGG Enrichment
Pathway
Adherens junction
Lysine degradation
Melanoma
Transcriptional misregulation in cancer
Cell adhesion molecules
Thiamine metabolism
Fc gamma R-mediated phagocytosis
Inositol phosphate metabolism
PI3K-Akt signaling pathway
Longevity regulating pathway
Arginine biosynthesis
Renin-angiotensin system
ECM-receptor interaction
Rap1 signaling pathway
Phosphatidylinositol signaling system
Prion disease
Asthma
African trypanosomiasis
Spliceosome
Mucin type O-glycan biosynthesis
0.00
0.05
0.10
RichFactor
GeneNumber
1
2
3
4
5
qvalue
0.5
0.4
0.3
0.2
0.1

Figure 3 Gel electrophoresis of total RNA mass.

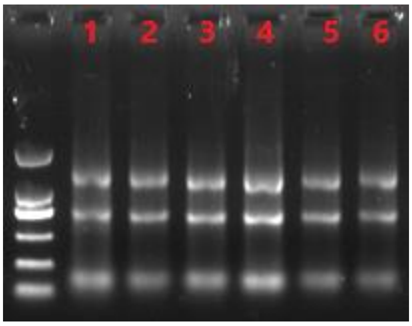

Figure 4 Principal component analysis diagram for broody chickens and normal laying hens.

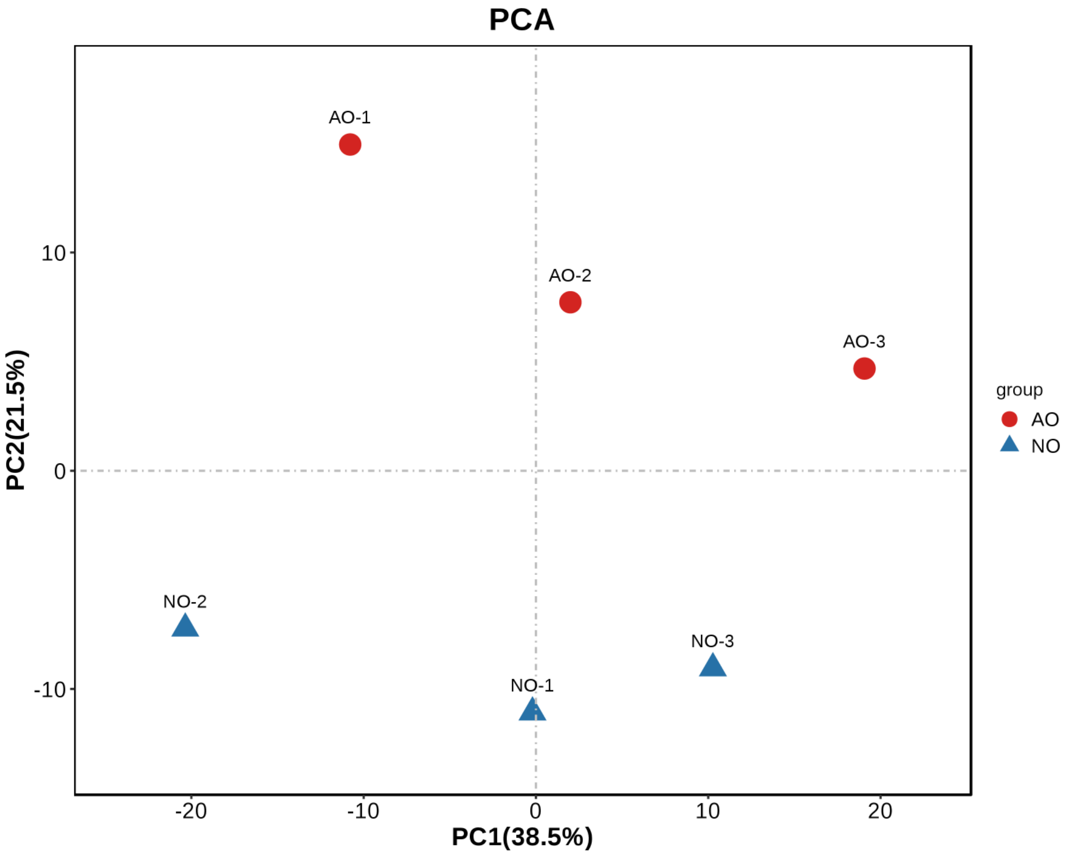

Figure 5 (A) Top 20 significantly changed GOs of ceRNAs in biological processes. (B) The top 20 pathways significantly associated with ceRNA transcripts.

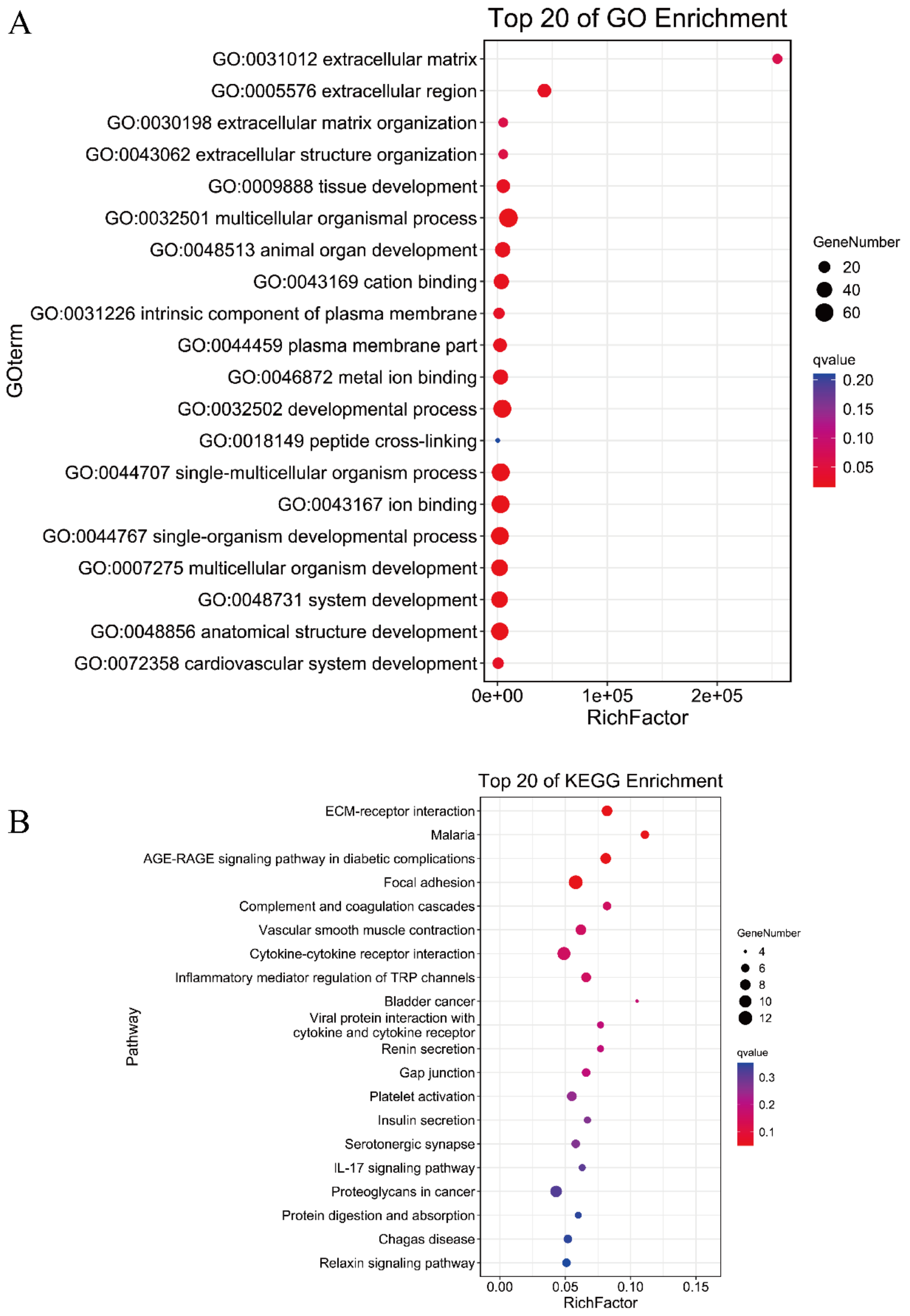

Figure 6 Violin diagram of miRNA expression

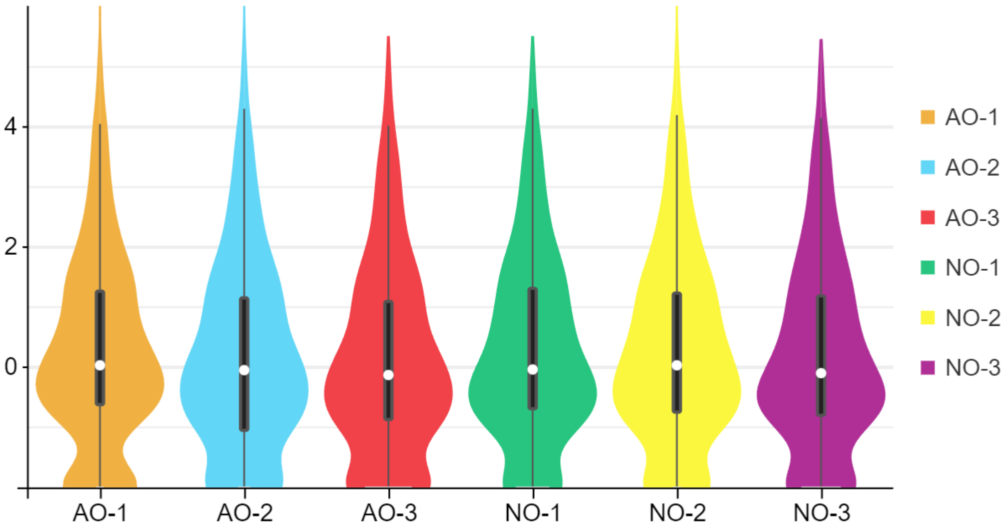

Supplement: Supplementary file 4 [file Image_1.pdf]
